# Flexible, affordable and environmentally sustainable solar vapor generation based on ferric tannate/bacterial cellulose composite for efficient desalination solutions†

**DOI:** 10.1039/d1ra05558e

**Published:** 2021-09-24

**Authors:** Thi Kieu Trang Nguyen, Quang Khai Dao, Daisuke Tanaka, Lien Ha Thi Nghiem, Minh Viet Nguyen, Zoom Hoang Nguyen, Tien Thanh Pham

**Affiliations:** Vietnam Japan University (VJU), Vietnam National University, Hanoi (VNU) Luu Huu Phuoc Street, Nam Tu Liem District Hanoi 100000 Vietnam pt.thanh@vju.ac.vn; Soft Matter and Biological Physics Center, Center for High Technology Development, Vietnam Academy of Science and Technology 18 Hoang Quoc Viet, Cau Giay Hanoi 100000 Vietnam; Department of Electrical and Electronic Engineering, National Institute of Technology, Oita College 1666 Maki Oita 870-0152 Japan; Institute of Physics, Vietnam Academy of Science and Technology 18 Hoang Quoc Viet Street, Cau Giay District Hanoi 100000 Vietnam; VNU–Key Laboratory of Advanced Materials for Green Growth, Faculty of Chemistry, University of Science, Vietnam National University Hanoi Vietnam

## Abstract

Desalination by solar steam generation (SSG) system is a green technology to produce pure water, which can address the issue of water scarcity. A novel photothermal material for the SSG system was fabricated by immersing bacterial cellulose (BC) sequentially into tannic acid (TA) and iron(iii) (Fe^3+^) solutions. Surface analysis of the resulting BC–TA–Fe^3+^ (BTF) material showed that coordination nanocomplexes between Fe^3+^ and hydroxyl groups of TA were formed on the surface of cellulose nanofibers. BTF material exhibited high sunlight absorption (∼95%), hydrophilic, self-cleaning properties, and excellent structural stability. SSG systems based on BTF had an evaporation efficiency of 91% and an evaporation rate of 1.56 kg m^−2^ h^−1^ under 1 sun illumination. Then, an efficient desalination device based on the larger-scale BTF material was fabricated to produce freshwater, the amount of freshwater per day was 5.6 kg m^−2^ on a sunny day. BTF material, thus, showed great potential in seawater desalination applications along with simple, versatile, scalable, and affordable fabrication methods.

## Abstract 2

The BTF material showed a great potential for seawater desalination applications along with a simple, versatile, scalable, and affordable fabrication method.

## Graphical abstract

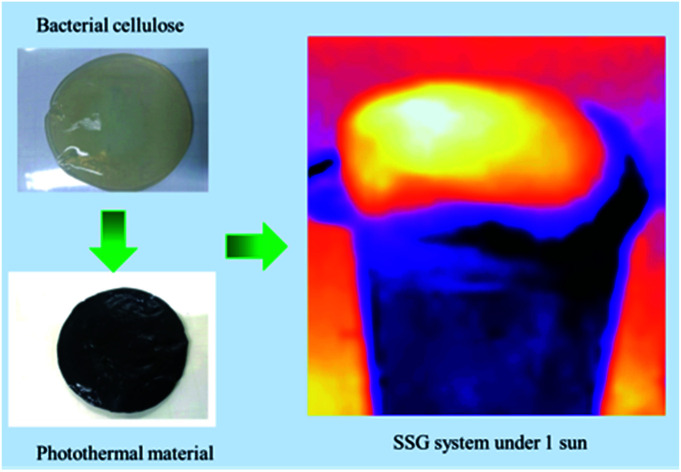

## 1. Introduction

Currently, the procedure of producing clean water from saline water utilizing solar steam generation (SSG) systems has captured increasing attention due to their outstanding features such as zero electricity consumption, sustainable, and eco-friendly technology.^1–3^ In SSG systems, heat converted from solar energy in the absorber is utilized to heat water at the evaporation surface. The water begins to evaporate when the accumulated heat reaches the enthalpy of water.^4^ Three key factors play a significant role for photothermal materials to improve the efficiency of SSG systems: (1) solar-thermal conversion capability, (2) water transportation rate, and (3) heat loss limitation.^2–4^ Many different types of materials have been utilized to fabricate the photothermal materials for SSG systems, which can absorb higher than 90% of the sunlight, such as metallic nanoparticles,^5,6^ semiconductors,^7,8^ porous polymer,^9,10^ and natural materials.^11,12^ Additionally, the photothermal materials have hydrophilic properties, porous structure, and capillary system, which are able to transfer quickly enough water from the bulk to the surface for the evaporation process.^4,8,13^ Besides, the materials have low thermal conductivity, which limits the conduction of heat to the surrounding environment.^14,15^ Therefore, SSG systems based on these photothermal materials demonstrated a high evaporation rate in the ranges of 1.0–1.7 kg m^−2^ h^−1^ under 1 sun illumination (1 kW m^−2^).^1–6^ Unfortunately, large-scale applications of SSG systems based on these materials have so far been still limited due to complicated fabrication methods, the high cost of raw materials, and low structural stability.^2,3,6,7,10^

Bacterial cellulose (BC) is versatile material composed of cellulose fibers having the size in the range of 100–200 nm that forms a three-dimensional scaffold, which has shown remarkably high Young's modulus value, high water uptake capacity, great crystallinity, a tremendous degree of polymerization and having fibers with high aspect ratio.^16,17^ The most notable properties of BC are the great porosity and huge total surface area. Therefore, it is a biocompatible material and thus suitable for sustainable fabrication processes, biomedical applications using BC composites.^18,19^ In addition, BC also has significant advantages as a photothermal material, such as a low thermal conductivity due to the porous structure (to reduce heat loss), hydrophilic properties the ability to quickly transfers water through the 3D network structure of BC.^20^ Many research groups have utilized the BC material to fabricate photothermal materials for the SSG system application in seawater desalination, such as the combination of BC and CuS semiconductor materials (BC@CuS),^21^ BC and polydopamine particles (BC@PDA),^22^ BC and biomass materials (BC@biomass).^23^ The BC-based SSG systems are highly efficient with the evaporation efficiency reaching 78–90% and water evaporation rate 1.2–1.44 kg m^−2^ h^−1^ under 1 sun illumination.^21–23^ Moreover, because of the structural stability and scalable feasibility, SSG systems based on BC composites have great potential for practical applications. Therefore, there is an urgent demand for novel BC-based photothermal materials with a simple fabrication process and eco-friendly input materials.

In this report, we propose a simple and affordable process to fabricate photothermal materials by treating BC with tannic acid (TA) and iron(iii) chloride (Fe^3+^) solutions. Structural analysis shows that the resulting BC–TA–Fe^3+^ (BTF) photothermal material had significant properties, such as high sunlight absorption ability, super hydrophilicity, high porosity, and low thermal conductivity. Additionally, this material exhibited significant durability in many different conditions such as salt water, acid, and alkaline solutions. Especially, it could be produced on a large scale in accordance with the requirements for photothermal materials applied in SSG systems.

## 2. Experimental section

### 2.1 Fabrication of bacterial cellulose

Tea was brewed by putting 11.3 g of green tea into 1.0 L of boiling water and allowed to stand for 20 minutes. The tea leaves were then removed, and 100 g of sucrose was dissolved in the tea and the whole solution was cooled to 37 °C. Then, kombucha's symbiotic colony of bacteria and yeast (SCOBY) was added. The resulting sweetened tea with the starting SCOBY was then covered with a cotton cloth and allowed to stand in a dark cupboard at 28 °C. The new kombucha BC formed at the air–water interface was carefully collected when it reached the needed thickness. After that, the collected kombucha BC was submerged in a 1.0 M aqueous sodium hydroxide solution thermostatic at 90 °C for 1 h. Then, BC was soaked in 1.5% (w/w) aqueous sodium hypochlorite (NaOCl) and allowed to stand at room temperature (23 °C) for 2.0 h. Then, BC was removed from the NaOCl bath and immersed 6 times in deionized water to remove entirely the cleaning compound. Finally, BC was stored in deionized water at 4 °C for subsequent experiments.

### 2.2 Fabrication of BTF material

Chemicals used to fabricate photothermal materials included tannic acid (C_76_H_52_O_46_; GHTECH; China) and iron(iii) chloride hexahydrate (FeCl_3_·6H_2_O; GHTECH; China). TA and Fe^3+^ solutions were prepared by dissolving 0.8 g TA in 200 ml deionized (DI) water and 0.4 g FeCl_3_·6H_2_O in 200 ml DI water, respectively. Photothermal materials based on BC were fabricated according to the following process:^24^ (1) BC (5 mm thickness, 4.5 cm diameter) was immersed in TA solution under stirring for 20 hours. Then, a BC–TA sample was rinsed with DI water to remove free TA particles. (2) BC–TA was immersed in Fe^3+^ solution with stirring for 2 hours. After that, a BC–TA–Fe^3+^ (BTF) material was rinsed with DI water to obtain BTF material, and then was freeze-dried for 6 hours.

### 2.3 Characterization of the BTF material

Cellulose nanofibers and a 3D network of BC and BTF materials were studied by scanning electron microscopy (SEM; JSM-IT100, JEOL Ltd, Japan) at an accelerated voltage of 10 kV. The surface functional groups of BC and BTF materials were analyzed using Fourier-transform infrared spectroscopy (FTIR; FTIR-4600; JASCO; Japan), energy dispersive spectroscopy (EDS; JED-2300, JEOL Ltd, Japan) and X-ray diffraction technique (XRD Mini Flex 600, Rigaku, Japan). The scattering, transmittance and reflectance spectra of the material were measured from 300 nm to 2500 nm using an infrared spectrometer (LAMBDA 950; PerkinElmer, Inc.; USA) attached to an integrating sphere. The absorbance A was calculated according to the formula *A* = 1 − *R* − *T*. The hydrophilic properties of BTF material were evaluated using water contact angle measurements. The mechanical properties of the materials were measured using a servo control system universal testing machine (Gotech Al 7000M; Gotech, Taiwan).

### 2.4 Seawater desalination test

The seawater evaporation performance was conducted using a class ABB solar simulator (94021A, Newport Corporation, USA) with an AM 1.5 G filter at an ambient temperature of 28 °C and a humidity of 40%. The BC photothermal material was attached to the commercial polystyrene (thickness of 20 mm) foam covered with a commercial cotton gauze (Tanaphar, Vietnam; thickness of 0.2–0.3 mm; mesh size of 2 mm × 2 mm), as illustrated in Fig. 4(a) (Fig. S1†). The samples were floated in a quartz beaker (4.5 mm in diameter) filled with seawater, that was taken from the Vietnam Sea. An electronic balance with a 0.01 mg resolution (Mettler Toledo, Switzerland) was utilized to record the changes in the mass of water during the water-evaporation experiments. Using a thermal imaging camera (FLIR C2; FLIR Systems, Inc.; USA), IR photographs of the samples were obtained, and the surface temperature distributions were monitored. Ion concentrations of seawater and desalinated water were measured by using SW-846 test method 6010D: inductively coupled plasma-optical emission spectrometry (ICP-OES; HORIBA, Japan) and Skalar SAN++ continuous flow analysis (CFA) analyzer (Skalar, Netherlands).

## 3. Results and discussion

**Fig. 1:**
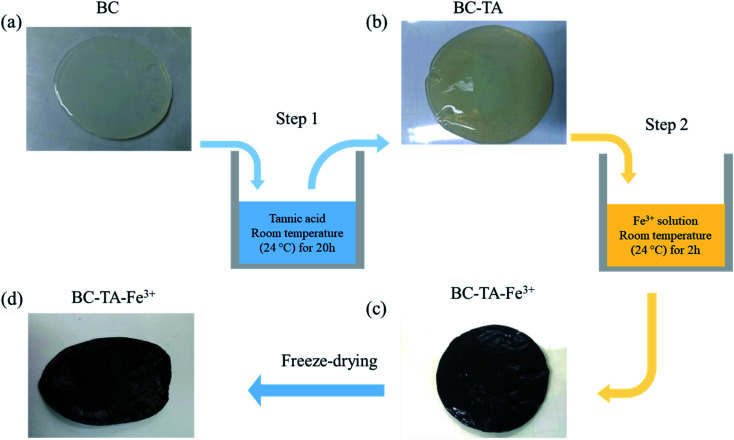
Schematic of the BTF material fabrication. (a) BC sample, (b) BC–TA made by treating the BC with TA in step 1, (c) BC–TA–Fe^3+^ (BTF), made by treating the BC–TA with Fe(iii) solution in step 2, (d) BTF after freeze-drying.

BC was fabricated by the fermentation process of the yeast and bacteria in an aqueous solution containing tea and sucrose at room temperature (28 °C) for 5–10 days (Fig. S1†). The thickness and area of the collected BC could easily be controlled depending on the growth time of BC in the solution. In this study, BC films were 5 mm thick and 4.5 cm in diameter. Fig. 1 describes the fabrication process of BTF based on BC. First, BC in white color (Fig. 1(a)) was submerged in the TA solution (Fig. S2a†). The surface of the BC–TA sample turned into light yellow, as shown in Fig. 1(b) because TA particles were attached to the BC surface *via* hydrogen bonds between TA molecules and cellulose molecules (Fig. S2b†). After treating BC–TA with Fe^3+^ solution, the color of the resulting BC–TA–Fe^3+^ material turned black (Fig. 1(c)), which was due to the formation of nanocomplexes consisting of Fe^3+^ and TA on the surface of cellulose nanofibers (Fig. S2c†). Finally, the material was freeze-dried to analyze structural and chemical properties as shown in Fig. 1(d). For comparison purposes, the BC material was directly immersed in Fe(iii) chloride solution for 2 h. However, the surface color of this sample only became light yellow due to the existence of Fe^3+^ ions. This result indicated that complexes between the Fe^3+^ ion and OH groups of the BC could not be formed.

### 3.1 Material structure and properties

**Fig. 2:**
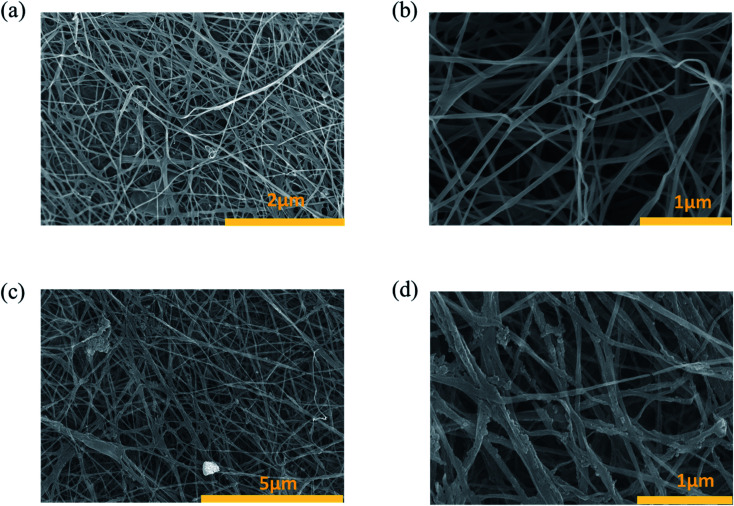
SEM images of BC and BTF materials: (a) BC sample shows cellulose fibers crisscrossing (bar: 2 μm), (b) high magnification SEM image of BC shows 20–50 nm cellulose nanofibers and ∼500 nm pores (bar: 1 μm) (c) SEM image of BTF material (bar: 5 μm), (d) high magnification SEM image shows clumps of nanocomplexes on cellulose nanofibers in BC (bar: 1 μm).

Fig. 2(a and b) are SEM images of the BC surface structure after the freeze-drying process. BC consisted of cellulose nanofibers, with a radius of 100–200 nm (Fig. 2(b)). Additionally, the BC structure was composed of many 3D network layers, which were a highly open microporous random 3D network of cellulose nanofibers.^21,22^ The 3D network of cellulose nanofibers was an important factor to increase the mechanical durability of BC, an essential feature for photothermal materials.^21^ The microporous structure of BC with a 3D network also played a key role in transferring water from the bulk to the air–water interface at the top of BTF materials to evaporate. SEM images of the BTF material are shown in Fig. 2(c and d). After being functionalized with TA and Fe^3+^ solutions, the layered structure and 3D network of the BC were still maintained. Moreover, nano-sized structures of 200–300 nm could be observed on the surface of cellulose nanofibers, which resulted from the formation of TA–Fe^3+^ nanocomplexes by the interaction between the hydroxyl groups of TA and Fe^3+^ ions.^25,26^ These nanostructures are not only attached to the cellulose nanofibers on the BC surface but also the nanofibers in the interior of BC. This was one of the reasons for the black color of BTF. The protonated phenolic group is not particularly good for the metal ions. However, once the phenolic group is deprotonated, and oxygen center is generated, it possesses a high charge density and can react with metal ions to form coordination complexes between oxygen anions and metal cations.^26^ The deprotonation that easily occurs in the phenolic group could be attributed to chemical compounds consisting of hydroxyl groups bonded directly to an aromatic ring. On the other hand, cellulose is a chemical compound consisting of hydroxyl groups bonded directly to sp^3^ hybridized carbon atom. In contrast to the hydroxyl groups, which are directly bonded to the aromatic ring, the polarizability of this group in cellulose molecules is quite low due to the absence of a conjugated system. Therefore, the iron(iii) coordination complex could not be easily formed with cellulose ligands.

**Fig. 3:**
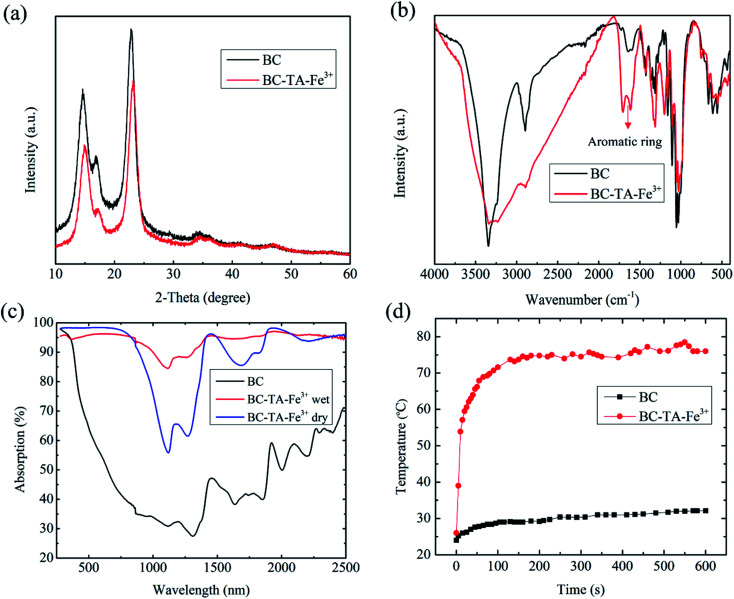
(a) XRD patterns of BC and BTF materials showing slight changes in the diffraction peaks, (b) FTIR spectra of BC and BTF materials show peak height difference at around 1610 cm^−1^, (c) absorption spectra of the BC and BTF in dry/wet conditions in the wavelength range of 200–2500 nm, (d) the maximum temperatures of BC and BTF materials under 1 sun illumination recorded by an IR camera.

The XRD analysis results of BC and BTF materials are shown in Fig. 3(a). The XRD pattern of BC exhibited three diffraction peaks at 2*θ* = 14, 17 and 22.35°, which had a contribution of diffractions corresponding to Iα and Iβ phases in the BC structure. On the other hand, the diffraction peaks in the XRD pattern of the BTF material slightly shifted to the right relative to the BC pattern, due to the presence of TA molecules with amorphous structures. No discernible peaks of crystalline structure for TA–Fe^3+^ nanocomplexes were observed in the XRD pattern, which would be similar to these in previous reports.^25,26^ The elemental composition of BC and BTF materials were analyzed by the EDS method, the results are shown in Table 1. The EDS of BC contained content a compound with a mixing ratio of C (44.73%): O (55.27%). Besides, Fe element (0.38%) could be found in the elemental composition of the BTF material, which showed Fe^3+^ ions being attached to BC surfaces by chelation between TA and Fe^3+^, which was formed on the cellulose nanofibers of BC.

**Table 1:** Chemical composition of BC, BC–TA, BTF materials

Sample	Composition (at%)		
	C	O	Fe
BC	44.73	55.27	—
BC–TA	51.8	48.2	—
BC–TA–Fe^3+^	52.27	44.8	0.38

Fig. 3(b) shows the FTIR spectrum of BC and BTF samples in the wavenumber range from 400 to 4000 cm^−1^. The FTIR spectrum of BC exhibited peaks at 3344, 2895, 1610–1725, 1313, 1107, 1030, and 612 cm^−1^. The peak at 3344 cm^−1^ was intrinsic to the OH stretching vibration of the hydroxyl groups, which existed in cellulose. The peaks in the range of 1610–1725 cm^−1^ characterized the C = O stretching vibration of the carbonyl group in cellulose. The peaks around 1107 and 1030 cm^−1^ were the characteristic peaks of C–O.^27^ After the functionalization of BC in TA and Fe^3+^ solutions, BC was attached by TA, including the OH functional groups of phenolic compounds and benzene. Therefore, the characteristic peaks of the aromatic ring at around 1610 cm^−1^ were detected clearly in the FTIR spectra of the BTF material. This result demonstrated that the BTF material had many hydrophilic functional groups, which were appropriate for the water transportation of BTF. Additionally, to investigate the hydrophilic properties of the BTF material, its contact angle measurement was conducted as shown in Fig. S4.† When 8 μl of water droplet reached the BTF surface, the water droplet can be permeated entirely into the BTF material in about 0.56 s (Fig. S4†), which also exhibited that the contact angle of the BTF surface was close to zero. Therefore, this result proved that the BC photothermal material had super hydrophilic properties, which was an important factor for water transportation from the bulk to the surface of BTF material.

The light absorption capacity of the photothermal material played an enormous role in improving the performance of SSG systems. The absorption of BC and BTF materials at wavelengths of 300–2500 nm was calculated from the measured scattering reflection, transmission spectra of the materials, which are shown in Fig. 3(c). It could be seen that the average absorption of BC was about 40–50% in the wavelength regions. On the other hand, the absorption of the freeze-dried BTF material can be reached approximately 96% in the wavelength range of 300–900 nm. However, the absorption of the material was unstable in the wavelength regions of 900–2500 nm, which was 60–95%. In the case of the BTF in wet conditions, the light absorption of the material was above 93% in the wavelength range of 300–2500 nm. The increase in the absorption of the BTF material in wet conditions could be attributed to water, which has significant absorption in the wavelength range of 1040–2500 nm. After treating BC with TA and Fe^3+^ solutions, the photothermal material based on BC could achieve high absorption ability, which was due to (1) nano-sized structures of ferric tannates, leading to the ligand-to-metal charge transfer band in the combination of compounds between the hydroxyl groups of TA and Fe^3+^,^24^ (2) the surface roughness and the 3D structure of the BC are shown in Fig. 2(d). The photothermal conversion ability of photothermal materials was investigated by placing BC and BTF materials under 1 sun illumination (1 kW m^−2^) for 600 s. The surface temperatures of these materials were recorded using the IR camera. Fig. 3(d) shows the changes in the maximum temperature of BC and BTF materials as a function of illumination time. With regard to the BC material, the surface temperature increased from 24 °C to 32 °C after 600 s of illumination, the slight changes in temperatures because of the low absorption ability in the wavelength range of 300–2500 nm. On the contrary, in the case of the BTF material, the surface temperatures rapidly increased from 24 °C to 71 °C in 100 s and became stable at 75 °C, which was the maximum temperature under 600 s of light illumination. Infrared images of the BTF surface corresponding to different times after light irradiation are shown in Fig. S5.† Therefore, BTF exhibited excellent photothermal conversion with high surface temperature and has a high potential for an efficient SSG system.

### 3.2 Seawater evaporation performances

**Fig. 4:**
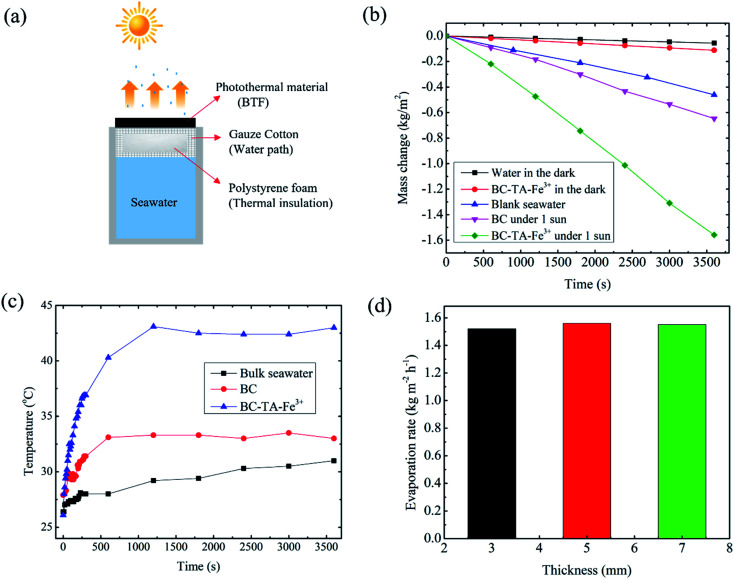
(a) Schematic of the seawater evaporation experiment, (b) the mass changes in seawater evaporation under dark conditions and 1 sun illumination for 60 min, respectively, (c) the maximum temperatures of BC and BFT materials based SSG surfaces, and bulk seawater during the seawater evaporation process, (d) the water evaporation rate of SSG system based on 3, 5, 7 mm-thick-BTF materials, respectively.

To evaluate the solar evaporation performance of BTF material, a bilayer SSG system was designed as shown in Fig. 4(a) and S6,† which consisted of 5 mm-thick BTF material (4.5 cm diameter) as the light absorber and a cotton gauze wrapped 20 mm thick polyethylene as a thermal insulator. The evaporation experiments were performed at room temperature (26 °C) and the average humidity ∼40%. The water evaporation efficiency of the material was measured by the volume reduction of water for 3600 s under 1 sun illumination (1 kW m^−2^). The evaporation rates of blank seawater and those of the SSG system utilizing BC and BTF materials were 0.461, 0.65, 1.56 kg m^−2^ h^−1^, respectively, as shown in Fig. 4(b). The evaporation rate of the BTF-based SSG system was 3.4 and 2.4 times higher than that of blank seawater and the BC material, respectively. The changes in the surface temperature of SSG systems during the evaporation of seawater under 1 sun condition is shown in Fig. 4(c). Infrared images of the BTF based SSG surface during the evaporation process are also shown in Fig. S7.† The initial temperature of the SSG surface before illumination was 23 °C. After light illumination, the surface temperature of the SSG rapidly grew in 400 s and became stable at approximately 41 °C for the BTF material. The surface temperature of the BTF could not increase higher because the transfer of water to the top surface of BFT was always kept stable through the 3D network structure in the BTF. This feature reduced heat loss due to heat radiation to the surrounding environment, leading to an increase in the water evaporation efficiency. Addinationly, Fig. 4(c) also illustrates maximum temperatures in the bulk water (black line) at 30 mm below the BTF surface under 1 sun illumination for 60 min. During the solar illumination, the water temperature increased slowly from 26.8 °C to 30.1 °C after 60 min. The obvious difference between the maximum temperature in the BTF material (41 °C) and temperature in the bulk water indicated that localized heating was achieved in the BTF surface (the interfacial evaporator) due to good thermal insulation by the low thermal conductivity of the BTF and polystyrene foam, leading to the high evaporation rate of the BTF-based SSG system.

To evaluate the effect of thickness of BTF material on the evaporation performance, water evaporation experiments for 3, 5, 7 mm-thick BTF materials were performed under 1 sun conditions, and the evaporation rate for each sample is shown in Fig. 4(d). The results show that the water evaporation rate of the SSG system based on the 5 mm-thick BTF material was 1.56 kg m^−2^ h^−1^, while those of the 3 and 7 mm-thick-materials were 1.54 and 1.56 kg m^−2^ h^−1^, respectively. Therefore, it could be seen that the thickness of the BC layer did not affect the water evaporation rate of the SSG much, which was about 1.56 kg m^−2^ h^−1^. Therefore, BC with a thickness of 3–5 mm was chosen for the fabrication of the BTF material in the SSG system. Under dark conditions, the evaporation rates of the blank seawater and the 5 mm-thick BTF material-based SSG were 0.06, 0.11 kg m^−2^ h^−1^, respectively, this result was used to calculate the evaporation efficiency of the SSG system. The evaporation efficiency was calculated by the following formula (1):^4,6^1
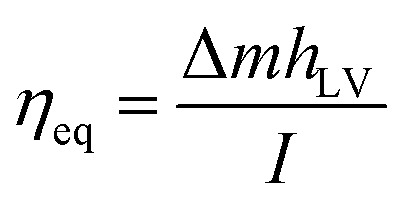
where Δ*m* is the water-evaporating amount under illumination; *h*_LV_ denotes the enthalpy of water vaporization (2260 J g^−1^), and *I* is the received power density of solar illumination. The water evaporation efficiency of the SSG system based on BC and BTF materials were 33.9% and 91%, respectively, which is comparable to those reported previously for SSG systems as shown in Table S1.†^21–23^ This high efficiency was achieved due to the remarkable properties of the BTF material such as super hydrophilic surface, the 3D network structure of nanocellulose fibers for rapid water transportation, high light absorption, reduction of heat loss by heat radiation to the ambient and conduction heat loss due to the low thermal conductivity of the BTF material (0.66 W m^−1^ K^−1^, as shown Fig. S8†).^20^

**Fig. 5:**
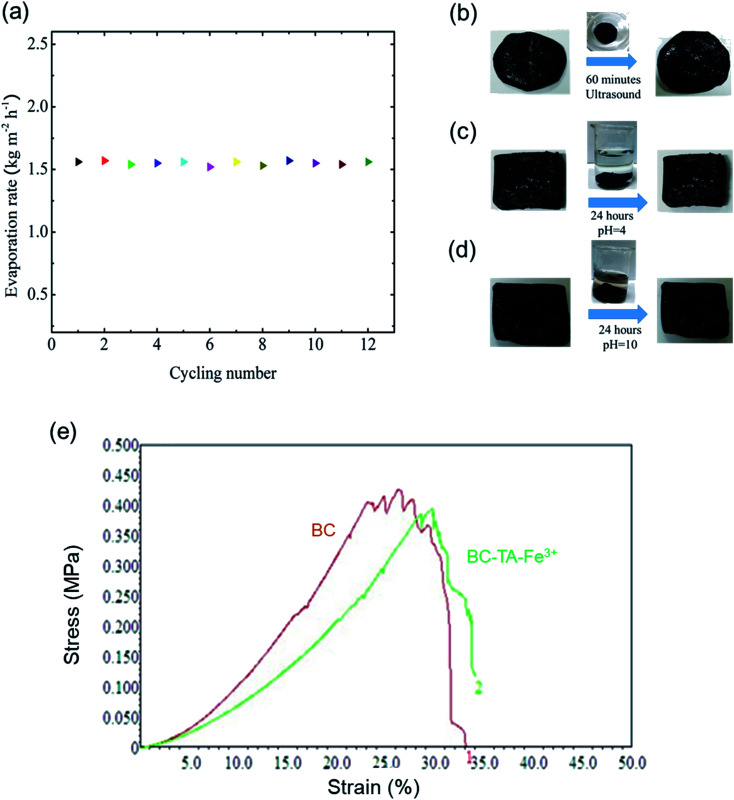
(a) Cycling stability of the SSG system under 1 sun illumination with 60 min irradiation for each cycle, the structural stability of BTF material: (b) ultrasonic vibration for 60 min, (c) immersion in acid solution (pH = 4), (d) aqueous alkali (pH = 10) for 24 hours, and (e) stress–strain curves for mechanical evaluation of the BC and BTF under wet conditions.

The structural stability of BTF material was also evaluated with a cycling test under 1 sun conditions and seawater with 60 min irradiation for each cycle. The SSG system based on BTF material exhibited a good stable mass change and the evaporation rate was 1.56 kg m^−2^ h^−1^, which was slightly changed, during the 12-cycle tests as shown in Fig. 5(a). In addition, the structural stability of the BTF material was also evaluated under various conditions such as ultrasonic vibration for 60 min, acid (pH = 4) and alkaline solution (pH = 10) for 24 hours. Fig. 5(b–d) exhibits the surface of the BTF samples after being treated. It could be seen that no changes were observed in the BTF surface, and they remained black. Additionally, the mechanical properties of BC and BTF materials were determined as shown in Fig. 5(e). The BC and BTF materials exhibited a tensile strength of 0.42 MPa and 0.4 MPa, respectively. It can be seen that the mechanical strength of both materials was almost equivalent, this high strength was due to the microporous random 3D network of cellulose nanofibers in the BC structure. Besides, this result also indicated that the TA–Fe^3+^ nanocomplexes on cellulose nanofibers did not affect the mechanical properties of the BTF material. Therefore, these results proved that the BTF material had great durability under many different conditions and was able to maintain evaporation performance for a long time. Besides, the self-cleaning properties of BTF material were also verified by placing 2 g of salt crystals on the surface of the BTF material, then the BTF material was placed on top of a beaker filled with seawater as shown in Fig. S9.† As could be seen from Fig. S9,† the number of salt crystals significantly decreased over time. After 180 min, almost no salt crystals could be observed on the BTF surface. The self-cleaning effect could be due to the phenomenon that water is transported from the bulk seawater to the surface dissolved salt crystals into ions, which diffused back down to the bulk seawater *via* the 3D network in the BC structure.^28^ These properties could reduce the number of salt crystals that had been accumulated during the seawater desalination process when water evaporation was not occurring (such as at night). The self-cleaning properties proved that the material could be used for a long time under real conditions. Specific results of salt ion concentration analysis after seawater desalination and performance of the SSG system under practical conditions would be reported in the future.

**Fig. 6:**
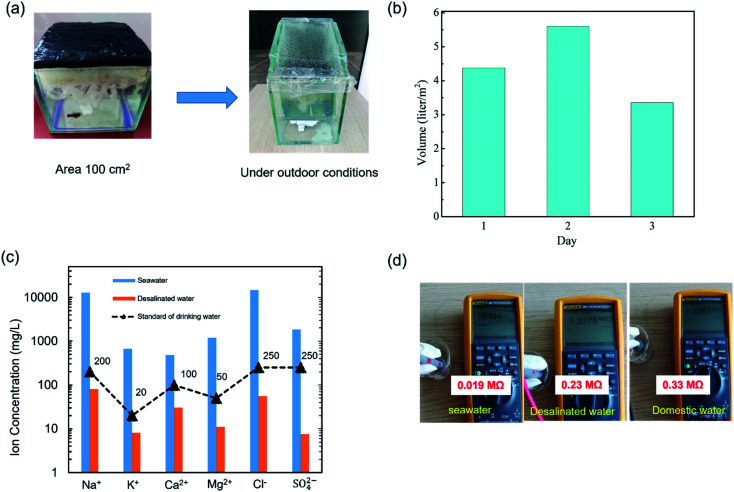
(a) A simple and low-cost solar desalination device, (b) performance of solar desalination under outdoor conditions (in Hanoi, Vietnam, May), (c) ion concentrations in seawater samples before and after the desalination process, and (d) electrical resistance of seawater, desalinated water, and domestic water.

To investigate the performance of the SSG system under real conditions, a simple and low-cost solar desalination device was prepared as shown in Fig. 6(a). The dimensions of the device were 250 mm high, 200 mm long and 120 mm wide and made from glass. The area of BTF material was 100 cm^2^ (Fig. 6(a)). The seawater was evaporated and condensed on the top glass surface under sunlight conditions from 9:00 AM to 5:00 PM in Hanoi, Vietnam for up to 3 days (May and June). The outdoor conditions (including solar intensity, temperature, and humidity) were recorded as shown in Fig. S10.†Fig. 6(b) exhibited the desalinated water from seawater per day under the above conditions. It was found that the produced pure water per day was 5.0–5.6 kg m^−2^ on sunny days and 3.0–3.9 kg m^−2^ on cloudy days, which was smaller than that under 1 sun illumination. Because the outdoor conditions were unstable (*e.g.* solar flux, humidity and temperature), the sunlight intensity propagating to the BTF surface decreased due to the top glass of the device. The ion concentrations in seawater and the desalinated water were measured and are shown in Fig. 6(c). The result indicated that the concentration of main ions (Ca^2+^, Mg^2+^, K^+^, Na^+^, Cl^−^, SO_4_^2−^) were significantly reduced after the desalination process, the concentration of Na^+^ and Cl^−^ were reduced from 12 932 mg L^−1^, 14 837 mg L^−1^ to 80.3 mg L^−1^, 55.3 mg L^−1^, respectively. Besides, the corresponding ion concentration of the desalinated seawater met the drinking water standards of the World Health Organization (WHO).^29^ Addinationly, to verify the purity of the desalinated water, the electrical resistances of domestic water, seawater, and the desalinated water were measured as shown in Fig. 6(d).^30,31^ The measured electrical resistances were 0.019 MΩ for seawater, 0.33 MΩ for domestic water, and 0.23 MΩ for the desalinated water. These results indicated that the concentrations of ions including cations and anions in the desalinated water were significantly reduced, the quality of the water was similar to domestic water and could be used as freshwater. The performances of the SSG based on the BTF materials under outdoor conditions showed great potential for practical applications of seawater desalination.

## 4. Conclusions

In conclusion, this research proposed the photothermal material based on the BC material for the SSG system by functionalizing BC in TA and Fe^3+^ solutions. The SSG system utilizing BTF material achieved the water evaporation rate of 1.56 kg m^−2^ h^−1^, and energy conversion efficiency of 91%. The high efficiency of BTF material was achieved due to outstanding characteristics of BTF materials such as light absorption over 91%, 3D network structure of nanocellulose fibers, which make the surface of the material super hydrophilic and rapidly transporting water in the structure, maintaining structural strength in seawater and various tough conditions. The performances of the SSG based on the BTF material in real conditions were evaluated, which produced 5.6 kg m^−2^ pure water per day on a sunny day. Additionally, BTF with a simple fabrication process, low cost, and eco-friendly input materials can be manufactured on a large scale, showing great potential for practical applications in SSG systems in the future.

## Conflicts of interest

There are no conflicts to declare.

## Supplementary Material

RA-011-D1RA05558E-s001
