# Supplementary material for: Flexible, affordable and environmentally sustainable solar vapor generation based on ferric tannate/bacterial cellulose composite for efficient desalination solutions
Source: RSC Adv. 2021 Sep 24;11(50):31641–9. doi: 10.1039/d1ra05558e (PMC9041557; doi:10.1039/d1ra05558e)
Supplement: RA-011-D1RA05558E-s001 [file RA-011-D1RA05558E-s001.pdf]

Electronic Supplementary Material (ESI) for RSC Advances.
This journal is © The Royal Society of Chemistry 2021

Supplementary Information

Flexible, Affordable and Environmentally Sustainable Solar Vapor Generation Based on Ferric Tannate/Bacterial Cellulose Composite for Efficient Desalination Solutions

Thi Kieu Trang Nguyen,[1] Quang Khai Dao,[2] Daisuke Tanaka,[3] Lien Ha Thi Nghiem,[4] Minh Viet Nguyen,[5] Zoom Hoang Nguyen,[2] Tien Thanh Pham,[1,]*

[1] *Vietnam Japan University (VJU), Vietnam National University, Hanoi (VNU), Luu Huu Phuoc Street, Nam Tu Liem District, Hanoi 100000, Vietnam*

[2] *Soft Matter and Biological Physics Center, Center for High Technology Development, Vietnam Academy of Science and Technology, 18 Hoang Quoc Viet, Cau Giay, Hanoi 100000, Vietnam*

[3] *Department of Electrical and Electronic Engineering, National Institute of Technology, Oita College, 1666 Maki, Oita, 870-0152, Japan*

[4] *Institute of Physics, Vietnam Academy of Science and Technology, 18 Hoang Quoc Viet Street, Cau Giay District, Hanoi 100000, Vietnam*

[5] *VNU–Key Laboratory of Advanced Materials for Green Growth, Faculty of Chemistry, University of Science, Vietnam National University, Hanoi, Vietnam.*

[*] *Email: pt.thanh@vju.ac.vn (Tien Thanh Pham)*

Keywords: Bacterial Cellulose; Photothermal materials; Seawater desalination; Solar Vapor Generation.

Process for the fabrication of BC

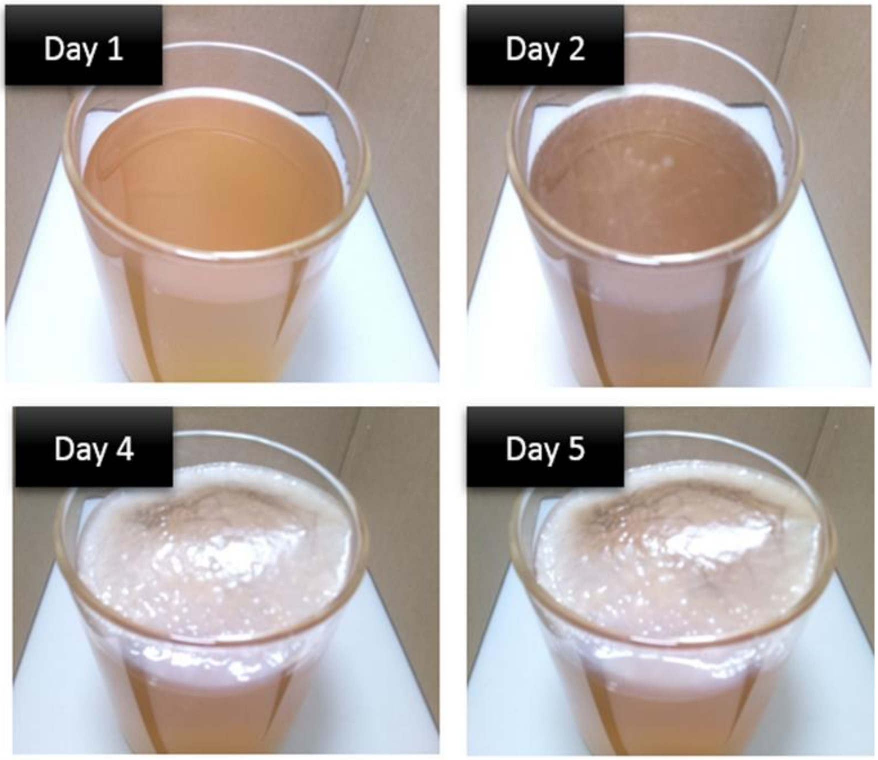

Figure S1. Fermentation process of the yeast and bacteria in the aqueous solution.

Schematic diagram of the chemical structures

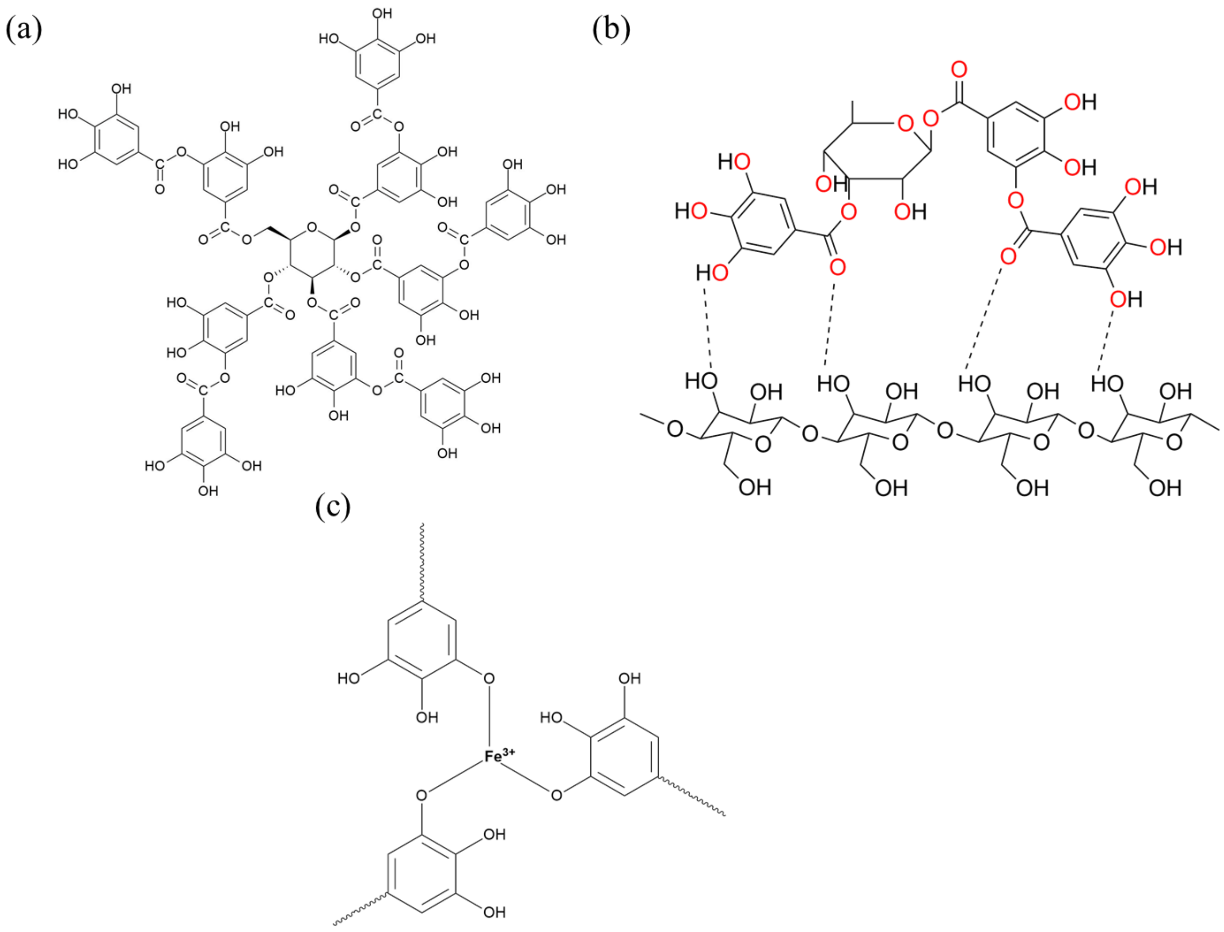

Figure S2. (a) Chemical structure of TA, (b) hydrogen bond between TA and cellulose, and (c) Fe^{3+}-OH coordination nanocomplexes.

BET analysis results

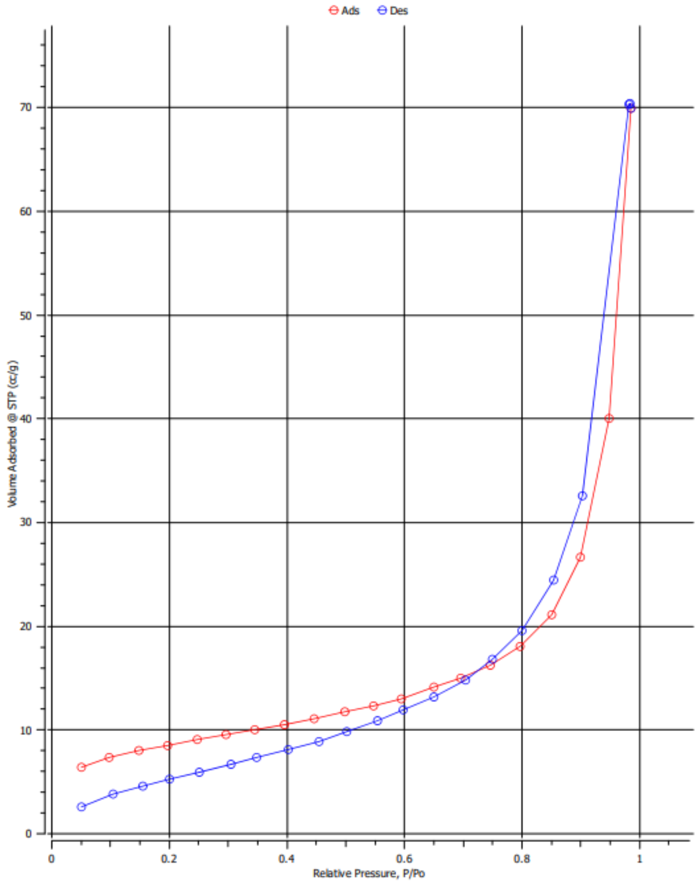

Figure S3. N2 absorption-desorpion insotherm of the BTF material.

BC exhibited large specific surface area (28.3 m^2 g^{-1}) as shown by BET analysis results in Figure S3.

The water contact angle of the BTF material

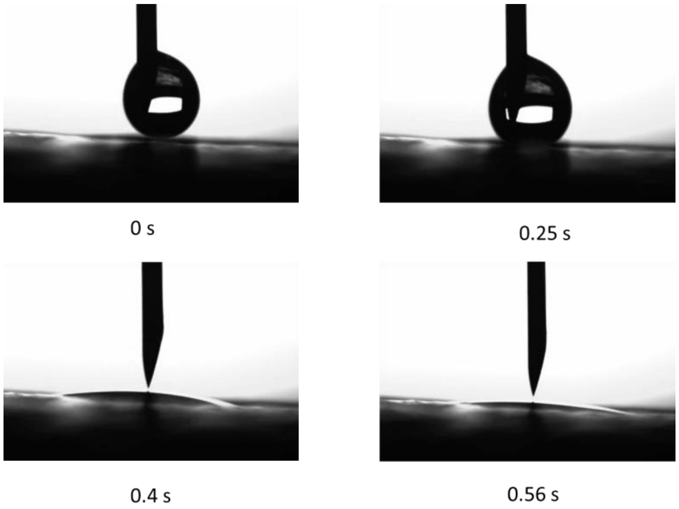

Figure S4. Water contact angle and water droplent absorption time of the BTF material.

IR camera images of the surface of BTF material under 1 sun illumination

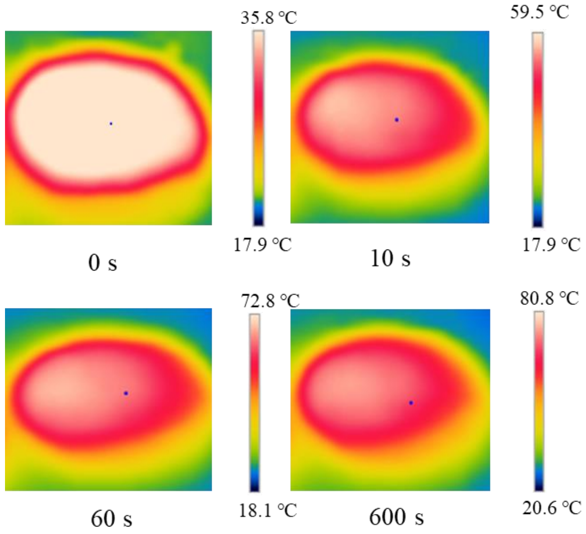

Figure S5. IR images of the surface of BTF material under 1 sun illumination over time.

Schematic illustration and photograph of the SSG system

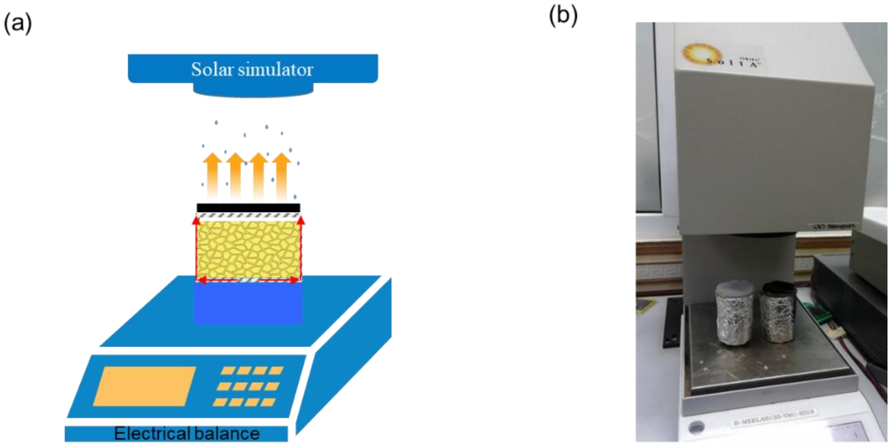

Figure S6. (a) schematic illustration and (b) photograph of the BTF-based SSG system.

The BC photothermal material (thickness of 5 mm, diameter of 4.5 cm) was attached to the commercial polystyrene (thickness of 20 mm) foam covered with a commercial cotton gauze (Tanaphar, Vietnam; thickness of 0.2–0.3 mm; mesh size of 2 mm × 2 mm), as illustrated in Figure 4a.

IR camera images of the surface of the SSG system based on BTF material under 1 sun illumination

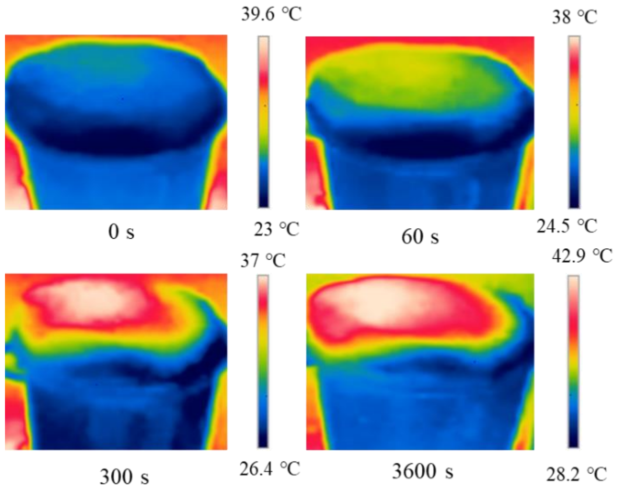

Figure S7. IR images of the surface of the SSG system under 1 sun illumination during evaporation process.

Thermal conductivity of the BTF material in wet condition

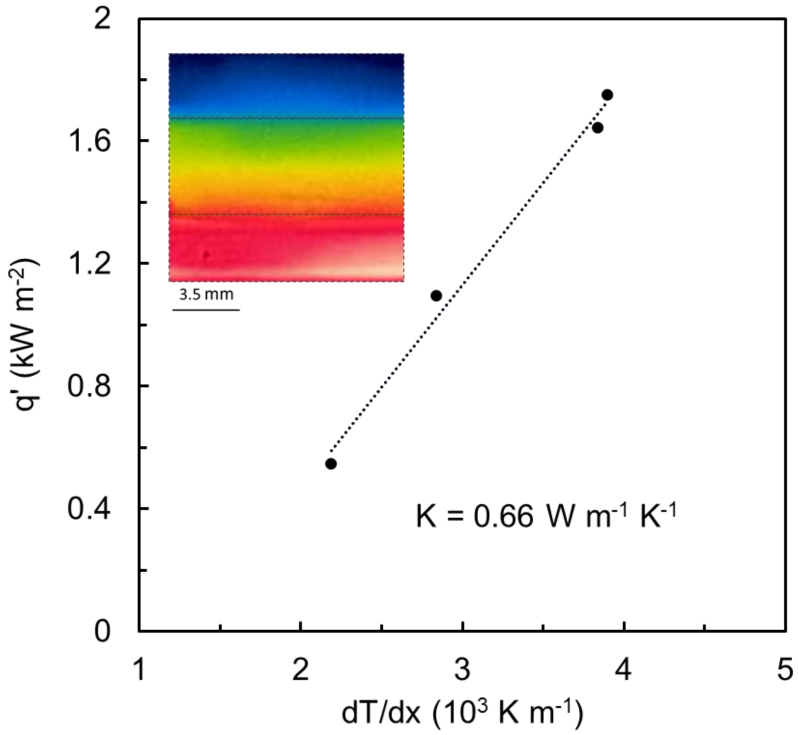

Figure S8. Thermal conductivity of the BTF material in wet state.

The thermal conductivities of the BTF materials in wet condition were measured by sandwiching the materials between two glass microscope slides (thickness of 3.5 mm), which were placed a hot plate and a glass slide with ice on top. The temperature distribution along the thickness was monitored using an IR camera.

The self-cleaning properties of the BTF material

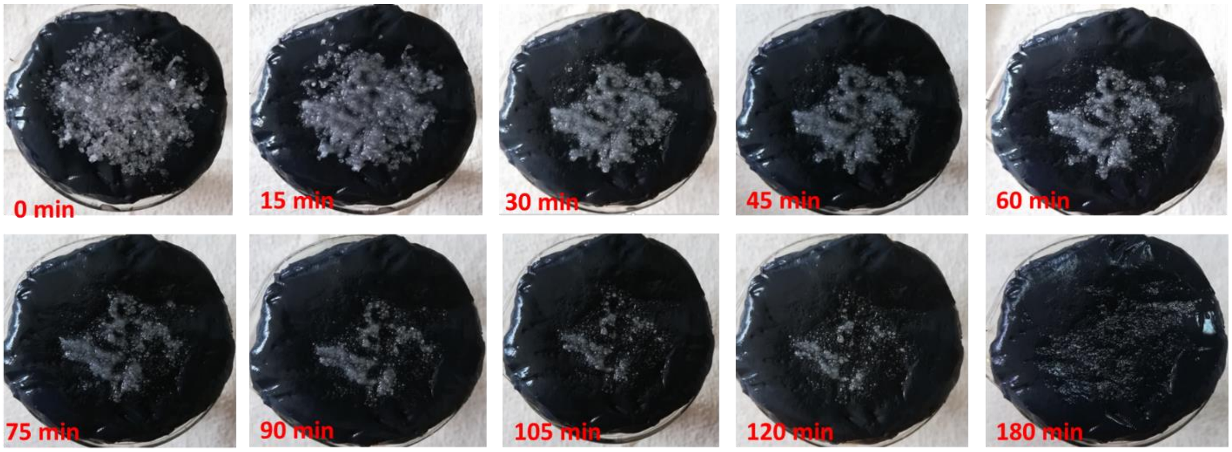

Figure S9. The self-cleaning process in the BTF surface over time.

The outdoor conditions

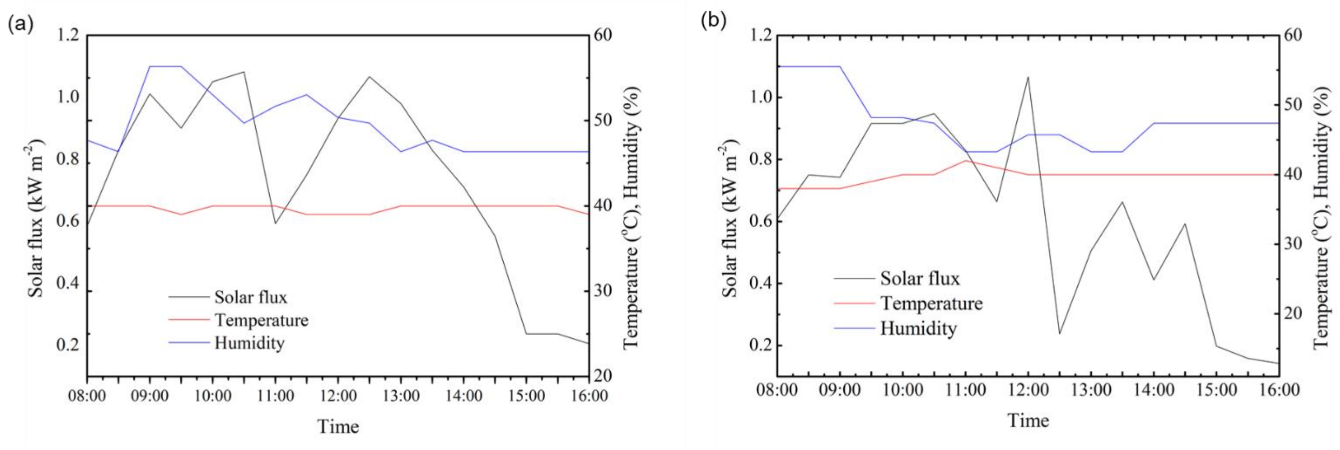

Figure S10. The solar intensity, humility, and temperature under during seawater desalination process under outdoor conditions for (a) day 2, and (b) day 1.

Table S1. Comparison of the performance of SSG systems in this work and previous.

Material	Evaporation rate, Efficiency	Limitation (price, fabrication method, scalability)	Reference
BTF material	1.56 kg m^{-2} h^{-1} 91% under 1 sun	Low price, simple fabrication, high scalability	This work
**Bacterial cellulose**			
Bacterial cellulose@Carbonized rice straw	1.2 kg m^{-2} h^{-1} 75.8 % under 1 sun	Low price, simple fabrication, medium scalability	1
Bacterial cellulose@PDA	1.13 kg m^{-2} $0.75h^{-1}$ 78% under 1 sun	Low price, simple fabrication, high scalability	2
Double-layer bacterial cellulose@Epichlorohydrin@Carbon black	1.58 kg m^{-2} h^{-1} 91.4% under 1 sun	Low price, simple fabrication, high scalability	3
Bacterial cellulose@GO	11.8 kg m^{-2} h^{-1} 83% under 10 sun (10 kW m^{-2})	Low price, simple fabrication, high scalability	4
Bacterial cellulose@CuS	1.44 kg m^{-2} h^{-1} 83.5% under 1 sun	Low price, simple fabrication, high scalability	5
**Carbonized biomass**			
Carbonized lotus seed	1.3 kg m^{-2} h^{-1} 80% under 1 sun	Low price, simple fabrication, medium scalability	6
Carbonized pomelo peel	1.39 kg m^{-2} h^{-1} 87.5% under 1 sun	Low price, simple fabrication, medium scalability	7
Carbonized corncob	1.358 kg m^{-2} h^{-1} 86.7% under 1 sun	Low price, simple fabrication, medium scalability	8
**Metallic nanoparticles**			
Au-NPs into nanofibre composite films	1.424 kg m^{-2} h^{-1} 83% under 1 sun	Expensive price, complicated fabrication, small	9
Pt/Au/TiO2-wood carbon	90.4% under 10 sun Low evaporation efficiency	Expensive price, complicated fabrication, small	10
Ni@C@ SiO2 core–shell nanoparticles	1.67 kg m^{-2} h^{-1} 91.2% under 1 sun	Expensive price, complicated fabrication, small	11
**Polymer**			
Multilayer PPy nanosheets	1.38 kg m^{-2} h^{-1} 88% under 1 sun	Low price, complicated fabrication, medium scalability	12

Polydopamine (PDA) and Pd nanoparticle (PDN) coated glass capillary array (GCA)	1.47 kg m^{-2} h^{-1} 89% under 1 sun	Expensive, complicated fabrication, large scalability	13

References:

1. Q. Fang, T. Li, Z. Chen, H. Lin, P. Wang and F. Liu, *ACS Appl. Mater. Interfaces* 2019, **11**, 10672-10679.

2. Q. Jiang, H. G. Derami, D. Ghim, S. Cao, Y.-S. Jun and S. Singamaneni, *J. Mater. Chem. A* 2017, **5**, 18397-18402.

3. N. Hu, Y. Xu, Z. Liu, M. Liu, X. Shao and J. Wang, *Carbohydr. Polym.* 2020, **243**, 116480.

4. Q. Jiang, L. Tian, K.-K. Liu, S. Tadepalli, R. Raliya, P. Biswas, R. R. Naik and S. Singamaneni, *Adv. Mater.* 2016, **28** (42), 9400-9407.

5. D. Zhang, M. Zhang, S. Chen, Q. Liang, N. Sheng, Z. Han, Y. Cai and H. Wang, *Desalination* 2021, **15**, 114899.

6. J. Fang, J. Liu, J. Gu, Q. Liu, W. Zhang, H. Su and D. Zhang, Chem. Mater. 2018, 30, 6217−6221.

7. X. Liu, D. D. Mishra, Y. Li, L. Gao, H. Peng, L. Zhang, and C. Hu, ACS Sustainable Chem. Eng. 2021, 9, 4571−4582.

8. T. Chen, H. Xie, X. Qiao, S. Hao, Z. Wu, D. Sun, Z. Liu, F. Cao, B. Wu, and X. Fang, ACS Appl. Mater. Interfaces 2020, 12 (45), 50397–50405.

9. M. Chen, Y. Wu, W. Song, Y. Mo, X. Lin, Q. He and B. Guo, Nanoscale 2018, 10, 6186.

10. M. Wang, P. Wang, J. Zhang, C. Li and Y. Jin, CheSusChem. 2019, 12, 467-472.

11. Z. Li, C. Wang, T. Lei, H. Ma, J. Su, S. Ling and W. Wang, Adv. Sustainable Syst., 2019, 3, 1800144.

12. X. Wang, Q. Liu, S. Wu, B. Xu and H. Xu, Adv. Mater. 2019, 31, 1807716.

13. Y. Fan, S. Wang, F. Wang, J. He, Z. Tian, H. Zhao, Z. Zhu, H. Sun, W. Lian and A. Li, J. Mater. Chem. A 2020, 8, 25904-25912.
